# Comparison of effectiveness and safety between uninterrupted direct oral anticoagulants with and without switching to dabigatran in atrial fibrillation ablation

**DOI:** 10.1002/joa3.12333

**Published:** 2020-03-18

**Authors:** Masahide Harada, Yuji Motoike, Yoshihiro Nomura, Asuka Nishimura, Masayuki Koshikawa, Kazuhiro Murayama, Yoshiharu Ohno, Eiichi Watanabe, Hideo Izawa, Yukio Ozaki

**Affiliations:** ^1^ Department of Cardiology Fujita Health University Toyoake Japan; ^2^ Joint Research Laboratory of Advanced Medical Imaging Fujita Health University Toyoake Japan

**Keywords:** anticoagulant, atrial fibrillation, catheter ablation, silent brain infarction, thromboembolism

## Abstract

**Introduction:**

Recent studies have demonstrated the feasibility of uninterrupted direct oral anticoagulants (DOACs) with a temporary switch to dabigatran (“dabigatran bridge”) for atrial fibrillation (AF) ablation. We compared the effectiveness and safety between uninterrupted DOACs with and without the “dabigatran bridge” in patients taking factor Xa inhibitors.

**Methods:**

AF patients on factor Xa inhibitors (rivaroxaban/apixaban/edoxaban) undergoing catheter ablation were eligible (n = 348). Brain MRI was performed within 2 days after the procedure to detect silent cerebral events (SCEs). Rivaroxaban/apixaban/edoxaban were uninterruptedly used in 153 patients (Group 1); these DOACs were switched to dabigatran on the day of AF ablation in 195 patients (Group 2). After propensity score matching, the unfractionated heparin (UFH) amount and the activated clotting time (ACT) kinetics during the procedure, the SCE incidence, and the follow‐up complications (30 days, thromboembolism and major/minor bleeding) in the two groups were compared.

**Results:**

Group 2 had higher initial ACT value and shorter time to optimal ACT (>300 seconds) than Group 1 (184 ± 36 s vs 145 ± 22 s, and 34 ± 29 s vs 43 ± 34 s, *P* < .05, respectively). Group 2 tended to require less amount of UFH to achieve optimal ACT than Group 1, but the total amount of UFH for the procedure was comparable. Group 2 had lower SCE incidence than Group 1 (16.2% vs 26.4%, *P* < .05). The prevalence of follow‐up complications was unchanged between the two groups.

**Conclusions:**

Switching to dabigatran on the day of AF ablation decreases preclinical thromboembolic events with similar bleeding risk to uninterrupted factor Xa inhibitors.

## Graphical abstract

Recent studies have demonstrated the feasibility of uninterrupted direct oral anticoagulants (DOACs) with a temporary switch to dabigatran (“dabigatran bridge”) for atrial fibrillation ablation. This study compared the effectiveness and safety between uninterrupted DOACs with and without the “dabigatran bridge” in patients taking factor Xa inhibitors. The results demonstrated that uninterrupted DOACs with "dabigatran bridge" decreased the incidence of MRI‐detected silent cerebral events with the similar bleeding risk to uninterrupted factor Xa inhibitors.

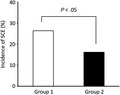

## 1 INTRODUCTION

Catheter ablation is a common intervention for atrial fibrillation (AF) but serious complications especially clinically apparent cerebral embolism and cardiac tamponade, remain a concern.^1^, ^2^ Therefore, periprocedural oral anticoagulation (OAC) should be optimized.

Direct oral anticoagulants (DOACs) became the practical standard for periprocedural OAC in AF ablation. In recent randomized trials, the uninterrupted use of DOACs demonstrated the incidence of major bleeding almost similar to, or even lower than, the uninterrupted use of vitamin‐K antagonist (VKA).^3^, ^4^, ^5^, ^6^ An expert consensus statement has recommended uninterrupted DOACs for periprocedural OAC in AF ablation.^7^

Idarucizumab, an antidote of direct thrombin inhibitor (dabigatran), is now available worldwide.^8^ However, andexanet alfa, an antidote of factor Xa inhibitors (rivaroxaban, apixaban, and edoxaban), is available in only limited countries.^9^ Serious bleeding may lead to worse outcomes in the presence of an irreversible anticoagulant. Dabigatran seems to be beneficial in countries without andexanet alfa, especially when used without interruption during the procedure. Recent studies have proposed the uninterrupted use of DOACs with a temporary switch to dabigatran (“dabigatran bridge”) in patients undergoing AF ablation and taking factor Xa inhibitors.^10^, ^11^ This OAC strategy seems to compensate for the drawbacks of the uninterrupted use of factor Xa inhibitors in the absence of andexanet alfa. However, this has not been fully evaluated in clinical practice.

In this study, we compared the effectiveness and safety between uninterrupted DOACs with and without “dabigatran bridge” in patients taking factor Xa inhibitors prior to AF ablation.

## 2 METHODS

### 2.1 Study population

This is a nonrandomized single center prospective study of AF patients undergoing catheter ablation at Fujita Health University from January 2015 to December 2018. Written informed consent was obtained from all patients undergoing catheter ablation for AF. Among them, those taking factor Xa inhibitors were enrolled. Baseline demographics/clinical information (AF type, past history, comorbidities, medications, etc) was obtained; CHADS_2_ and CHA_2_DS_2_‐VASc scores were calculated. Laboratory examinations (creatinine and brain natriuretic peptide, etc) and transthoracic echocardiography (ejection fraction and left atrial dimension, etc) were performed before catheter ablation. Transesophageal echocardiography (TEE) was performed one day before the procedure, and patients with left atrial appendage thrombus detected by TEE were excluded. Patients with creatinine clearance (CrCl, calculated by Cockcroft‐Gault formula) <15 mL/min and those on hemodialysis were excluded from the study. Patients with mechanical valves were also excluded.

### 2.2 Oral anticoagulation therapy

All patients were treated with rivaroxaban (15 mg/10 mg q.d.), or apixaban (5 mg/2.5 mg b.i.d.), or edoxaban (60 mg/30 mg q.d.) for ≥4 weeks before AF ablation; patients treated with dabigatran were excluded. Of note, 15 (10) mg q.d. of rivaroxaban was officially approved as regular (low) doses for Japanese AF patients due to their smaller bodies/different pharmacokinetics.^12^ Rivaroxaban and edoxaban, once‐daily drugs, were administered in the morning. Approved dose criteria were specific to each DOAC according to the patient's renal function, weight, age, and concomitant medications, as indicated in the approved package inserts (PIs).^13^, ^14^ In all patients, the initial choice/dose of anticoagulants was decided by the physicians/cardiologists who diagnosed AF; the anticoagulants were continued before ablation without change, interruption, and heparin bridging. Patients treated with DOACs who did not follow the PIs in each DOAC were excluded; eligible patients received DOACs at an appropriate dose.

**FIGURE 1:**
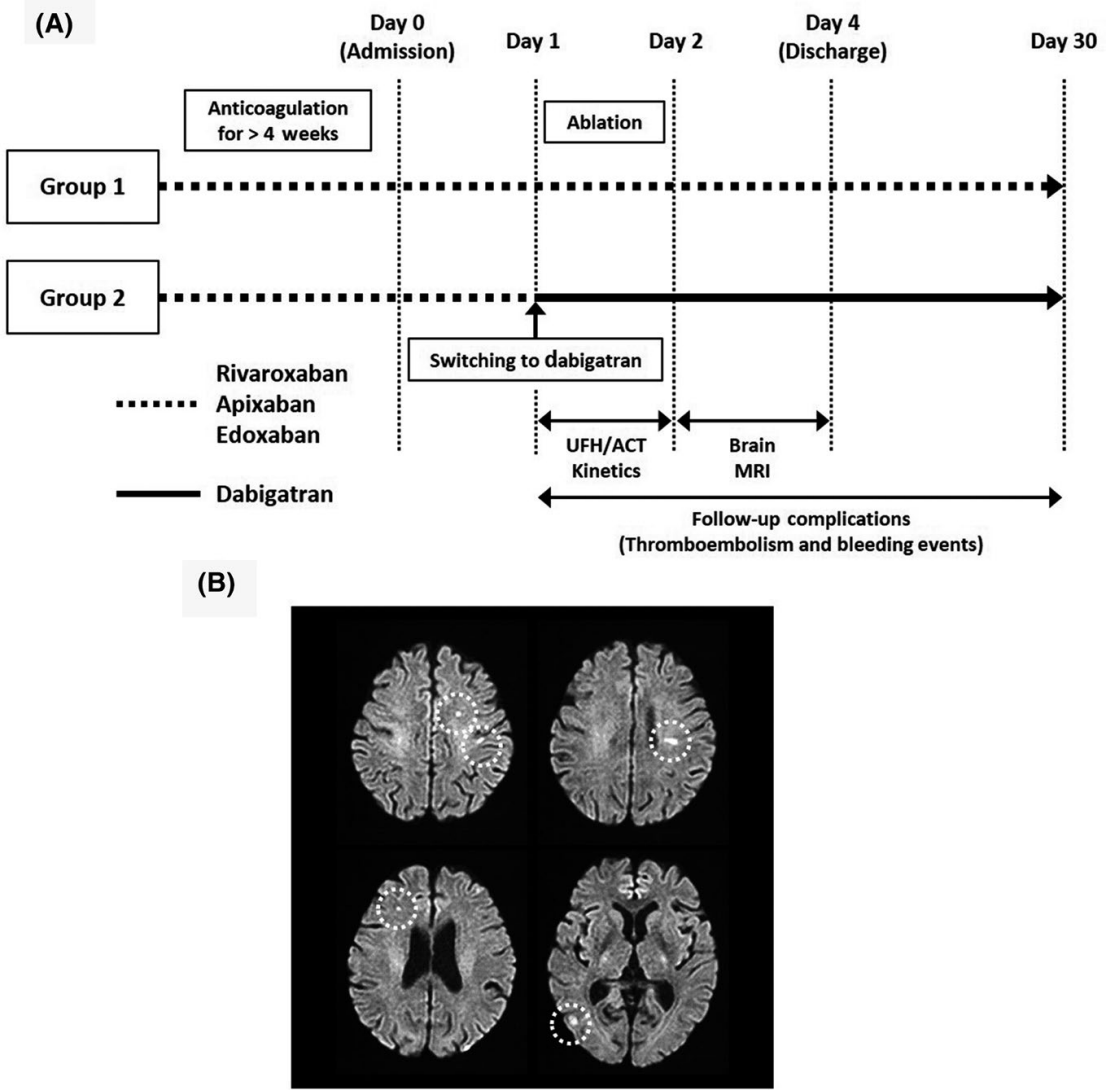
A, Schematic diagram of anticoagulation protocol in each group. B, Example of silent cerebral events (SCEs) detected in the brain MRI in a patient. White dotted circles indicate SCE lesions. ACT, activated clotting time; UFH, unfractionated heparin

Before May 2017, rivaroxaban, apixaban, or edoxaban was uninterruptedly used during the procedure (Group 1). Idarucizumab became available in May 2017, but andexanet alfa has not yet been approved here in Japan. For safety reasons, dabigatran was preferred for use at least during AF ablation in the case of adverse bleeding. Thus, since May 2017, rivaroxaban, apixaban, and edoxaban were continued until one day before AF ablation (the day of hospital admission) and then the anticoagulants were switched to dabigatran on the day of the procedure (Group 2). If a patient's CrCl was less than 50 mL/min, the low dose (dabigatran 110 mg b.i.d.) was selected. Dabigatran was maintained after the procedure. Figure 1A shows the schematic diagram of anticoagulation protocol for each group.

A proton pump inhibitor was prescribed in all patients and was continued at least for the 30‐day follow‐up period after the procedure to minimize the risk of esophageal injury.

### 2.3 Ablation procedure

In paroxysmal AF patients, pulmonary vein isolation (PVI) was performed using either cryoballoon ablation (CBA, single short freezing for 180 seconds in each PV) or radiofrequency catheter ablation (RFCA) in the first session. RFCA was selected in patients with common PV or large PV ostium (>28 mm) based on the LA/PV anatomy evaluated by cardiac‐computed tomography imaging before the procedure. In persistent AF patients, only RFCA was used for PVI in the first session. In all patients, only the PVI strategy was used for the first AF ablation. In the second session for both types of AF, the incomplete line of PVI was repaired with RFCA if necessary, and additional posterior wall isolation (linear ablation at the roof and bottom between left and right PV) and superior vena cava isolation were performed.

The CBA procedure was achieved using electroanatomical mapping (EnSite NavX, Abbott) and fluoroscopic guidance to position the cryoballoon catheter. In the RFCA procedure, PVI was achieved using a focal “point‐by‐point” catheter approach, delivering radiofrequency energy to the cardiac tissue with irrigation tip catheters (THERMOCOOL SMARTTOUCH^®^ SF, Biosense Webster [target contact force: 10‐20 g, RF time: 30‐60 seconds, irrigation flow rate: 8 mL/min for ≤30W, 15 mL/min for >30W, power control mode], or FlexAbility^TM^, Abbott [RF time: 30‐60 seconds, irrigation flow rate: 10 mL/min for <38°C, 13 mL/min for ≥38°C, temperature control mode]). RFCA lesion sets encircled the PV antra using electroanatomical mapping (CARTO3, Biosense Webster or EnSite NavX, Abbott) and fluoroscopy guidance.

### 2.4 Anticoagulation during procedure

Baseline activated clotting time (ACT) was measured before a first injection of unfractionated heparin (UFH). Then, a bolus of UFH (80‐120 IU/kg) was administered. ACT was measured every 10‐20 minutes after the first UFH shot, and additional UFH boluses (20‐60 IU/kg) were administered before transseptal puncture to reach optimal ACT (>300 seconds) for AF ablation.^15^ The time from the baseline ACT measurement until reaching optimal ACT and the amount of UFH to achieve optimal ACT was calculated. During the procedure, ACT was measured every 20‐30 minutes; additional UFH boluses were administered to maintain the ACT > 300 s. The total amount of UFH required for the procedure was also calculated.

### 2.5 Brain MRI

Brain MRI was performed in all patients within 2 days after the procedure using a 1.5 Tesla (T) scanner (Achieva 1.5T Nova Dual; Philips Healthcare) with an 8‐channel brain coil, or a 3T scanner (Ingenia 3T; Philips) with the dS head coil, or a Vantage Titan 3T (Canon Medical Systems Corporation) with a 16‐ or a 32‐channel coil to defect preclinical silent cerebral events (SCEs). In each patient, axial diffusion‐weighted imaging was performed using single‐shot, spin‐echo, echo planar imaging with two *b* values of 0 and 1000 s/mm^2^ and three diffusion directions. Other scan parameters were as follows: repetition time/echo time 3600‐5100/83‐98 msec, 112‐176 × 128‐256 matrix, 288‐512 × 288‐512 reconstruction matrix, 220 × 220 mm field of view, slice thickness 5.0 mm, slice gap 1.0 mm, and 1‐4 excitations. The apparent diffusion coefficient map (ADC‐map) was obtained to prevent overdetection of T2 shine‐through effects on diffusion‐weighted imaging.

The definition for diagnosing SCE was based on the detection of new hyperintense lesions of the diffusion‐weighted MRI with hypointense findings of the ADC‐map according to a neuroimaging expert's recommendation (Figure 1B).^16^ MRI images were independently evaluated by certified radiologists in a blinded manner. A neurological examination was performed on hospital admission and after the ablation procedure by certified neurologists or certified physicians blinded to the MRI findings. Neurological dysfunction was evaluated using the National Institutes of Health Stroke Scale (NIHSS) and the modified Rankin Scale (mRS).

### 2.6 Clinical follow‐up

Follow‐up complications of thromboembolic and bleeding events were assessed within 30 days after the procedure. Thromboembolic events included symptomatic transient ischemic attack (TIA), cerebrovascular accidents, and systemic embolic events. Major bleeding events were defined by the International Society of Thrombosis and Haemostasis (ISTH).^17^ Clinically relevant bleeding events that did not fulfill ISTH criteria for major bleeding events were defined as minor bleeding events.

### 2.7 Statistical methods

Continuous variables, represented as mean ± standard deviation, were compared using unpaired *t* tests. Categorical data, expressed as frequencies and percentages, were compared using chi‐square tests.

Because of the heterogeneity of the groups’ sample sizes and patient characteristics, propensity score matching was performed by the nearest neighbor technique to reduce the effect of potential confounding factors. Propensity scores were estimated from a logistic model and matched using a caliper width equal to 0.2 of the standard deviation of the logit of propensity score. Variables that potentially affect the incidence of SCE, thromboembolisms, and bleeding events were selected; predictors for propensity score matching were age, sex, type of AF, CHADS_2_ score, left‐atrial diameter, and type of procedure (RFCA or CBA).

All tests were two sided, and a *P* < .05 was considered statistically significant. Statistical analyses were performed using JMP11 (SAS Institute).

## 3 RESULTS

### 3.1 Patient characteristics

In this study, 348 patients treated with rivaroxaban (15 mg/10 mg q.d., n = 125/4), apixaban (5 mg/2.5 mg b.i.d., n = 73/9), and edoxaban (60 mg/30 mg q.d., n = 89/48) were eligible. These factor Xa inhibitors were uninterruptedly used in 153 patients (Group 1). On the other hand, these drugs were switched to dabigatran on the day of AF ablation in 195 patients (Group 2). In Group 2, some patients felt mild dyspepsia and gastric hypomotility but no patients discontinued dabigatran at least during the follow‐up period. After propensity score matching, 272 patients were selected for comparison (Group 1: n = 136 [rivaroxaban: n = 48, apixaban: n = 41, edoxaban: n = 47], Group 2: n = 136 [rivaroxaban: n = 50, apixaban: n = 24, edoxaban: n = 62]). Table 1 summarizes patients’ characteristics before and after propensity score matching.

**TABLE 1:** Patient characteristics before/after propensity score matching

	Before propensity score matching			After propensity score matching		
	Group 1 (n = 153)	Group 2 (n = 195)	*P* value	Group 1 (n = 136)	Group 2 (n = 136)	*P* value
Age, y	62.9 ± 12.3	64.7 ± 11.0	.151	64.7 ± 10.5	63.9 ± 11.0	.551
Male, n (%)	105 (69)	141 (72)	.455	92 (68)	95 (70)	.695
BMI, kg/m^2^	23.4 ± 3.3	23.7 ± 3.6	.338	23.2 ± 3.3	23.5 ± 3.9	.526
Persistent AF, n (%)	46 (30)	67 (34)	.395	42 (31)	40 (29)	.792
CHADS_2_ score (pts)	1.16 ± 1.16	1.15 ± 1.09	.490	1.15 ± 1.1	1.17 ± 1.18	.914
CHA_2_DS_2_‐VASc score (pts)	2.07 ± 1.64	2.01 ± 1.51	.347	2.01 ± 1.49	2.14 ± 1.67	.514
CHF, n (%)	30 (19)	34 (17)	.604	24 (18)	22 (16)	.746
HT, n (%)	65 (42)	104 (53)	.044	59 (43)	67 (49)	.109
Age ≥75, n (%)	26 (17)	38 (19)	.550	26 (19)	24 (18)	.776
DM, n (%)	24 (16)	29 (15)	.834	20 (15)	21 (15)	.865
Stroke/TIA, n (%)	16 (10)	10 (5)	.061	16 (11)	8 (7)	.085
Vascular disease, n (%)	8 (5)	13 (7)	.574	8 (6)	7 (5)	.791
Blood test and UCG						
Cr, mg/dL	0.82 ± 0.18	0.83 ± 0.21	.678	0.82 ± 0.19	0.86 ± 0.51	.361
CrCl, mL/min	82.7 ± 26.1	83.9 ± 30.1	.680	79.4 ± 22.8	83.9 ± 29.4	.154
BNP, pg/mL	122 ± 150	140 ± 178	.321	129 ± 157	133 ± 187	.836
EF, %	56.7 ± 9.6	56.7 ± 8.9	.985	56.8 ± 9.3	57.2 ± 8.1	.758
LAD, mm	37.4 ± 7.2	38.9 ± 6.7	.040	37.9 ± 6.7	37.9 ± 6.7	.481
Medication						
β‐blocker, n (%)	71 (46)	94 (48)	.739	61 (45)	67 (50)	.466
Antiplatelets, n (%)	13 (8)	14 (7)	.649	12 (9)	9 (7)	.495
AAD, n (%)	24 (16)	27 (14)	.631	23 (17)	17 (13)	.304
Low dose NOAC, n (%)	23 (15)	38 (19)	.276	22 (16)	33 (24)	.096
Ablation procedure						
Cryoballoon, n (%)	57 (37)	73 (37)	.972	51 (37)	52 (38)	.901
Additional linear ablation, n (%)	18 (12)	23 (12)	.993	16 (12)	15 (11)	.849

Abbreviations: AAD, antiarrhythmic drug; ACT, activated clotting time; AF, atrial fibrillation; BMI, body mass index; CHF, congestive heart failure; Cr, creatinine; CrCl, creatinine clearance; DM, diabetes mellitus; EF, ejection fraction; HT, hypertension; LAD, left atrial diameter; SCE, silent cerebral event; TE, thromboembolism; TIA, transient ischemic attack; UCG, ultrasonic echocardiography UFH, unfractionated heparin.

### 3.2 UFH amount and ACT kinetics

**FIGURE 2:**
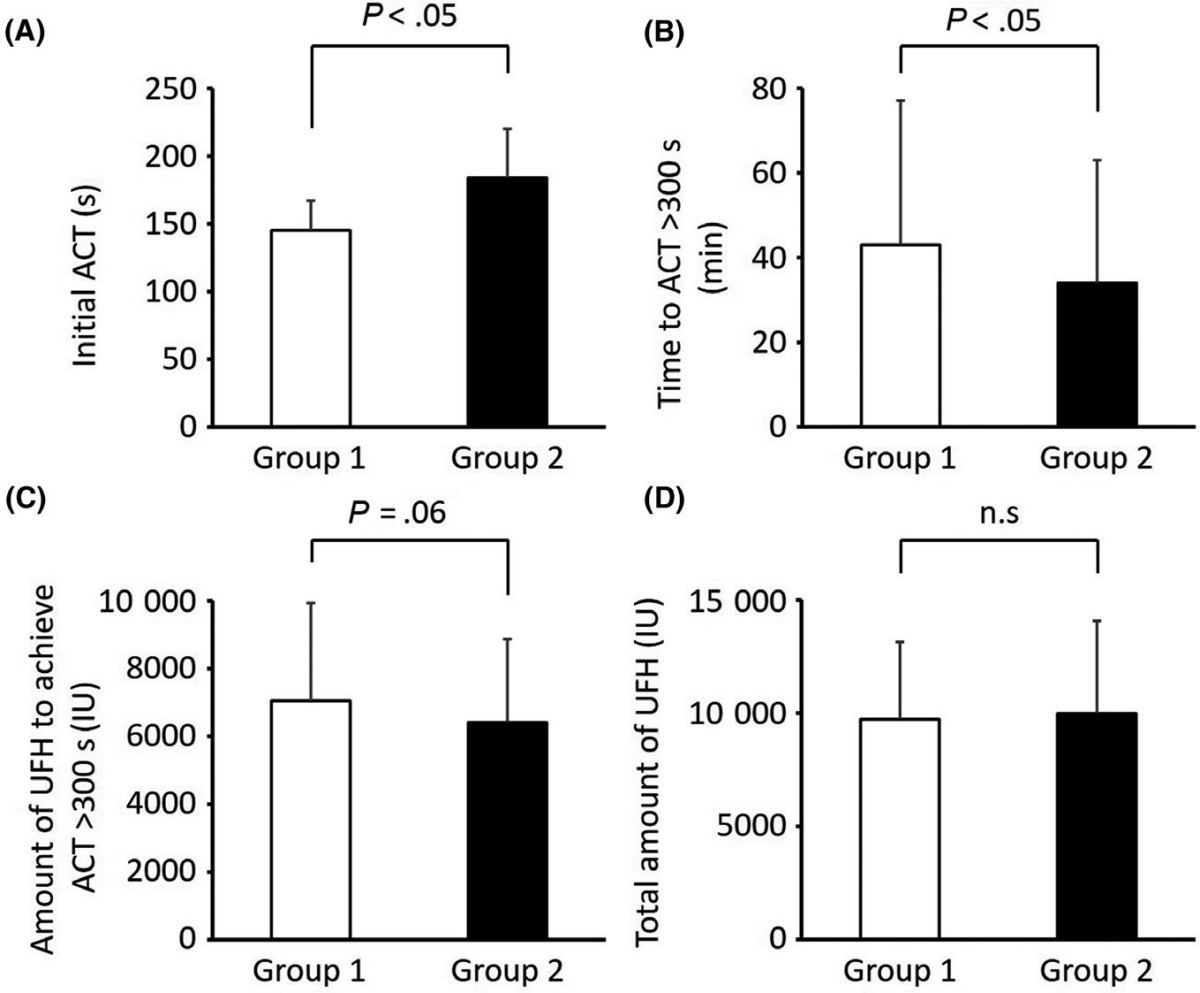
A, Initial ACT value before the first heparin shot. B, Time to reach optimal ACT (>300 s) C, Amount of UFH required to achieve the optimal ACT. D, Total amount of UFH. ACT, activated clotting time; UFH, unfractionated heparin

Initial ACT before first heparin injection significantly increased in Group 2 (184 ± 36 s vs 145 ± 22 s, Figure 2A), and the time to reach optimal ACT (>300 seconds) decreased in Group 2 (34 ± 29 s vs 43 ± 34 s, Figure 2B). The amount of UFH required to achieve the optimal ACT tended to decrease in Group 2 (Figure 2C), but the total amount of UFH between the two groups remained unchanged (Figure 2D). Group 2 achieved optimal ACT more quickly than Group 1; this finding is associated with the higher initial ACT value.

### 3.3 SCE

**FIGURE 3:**
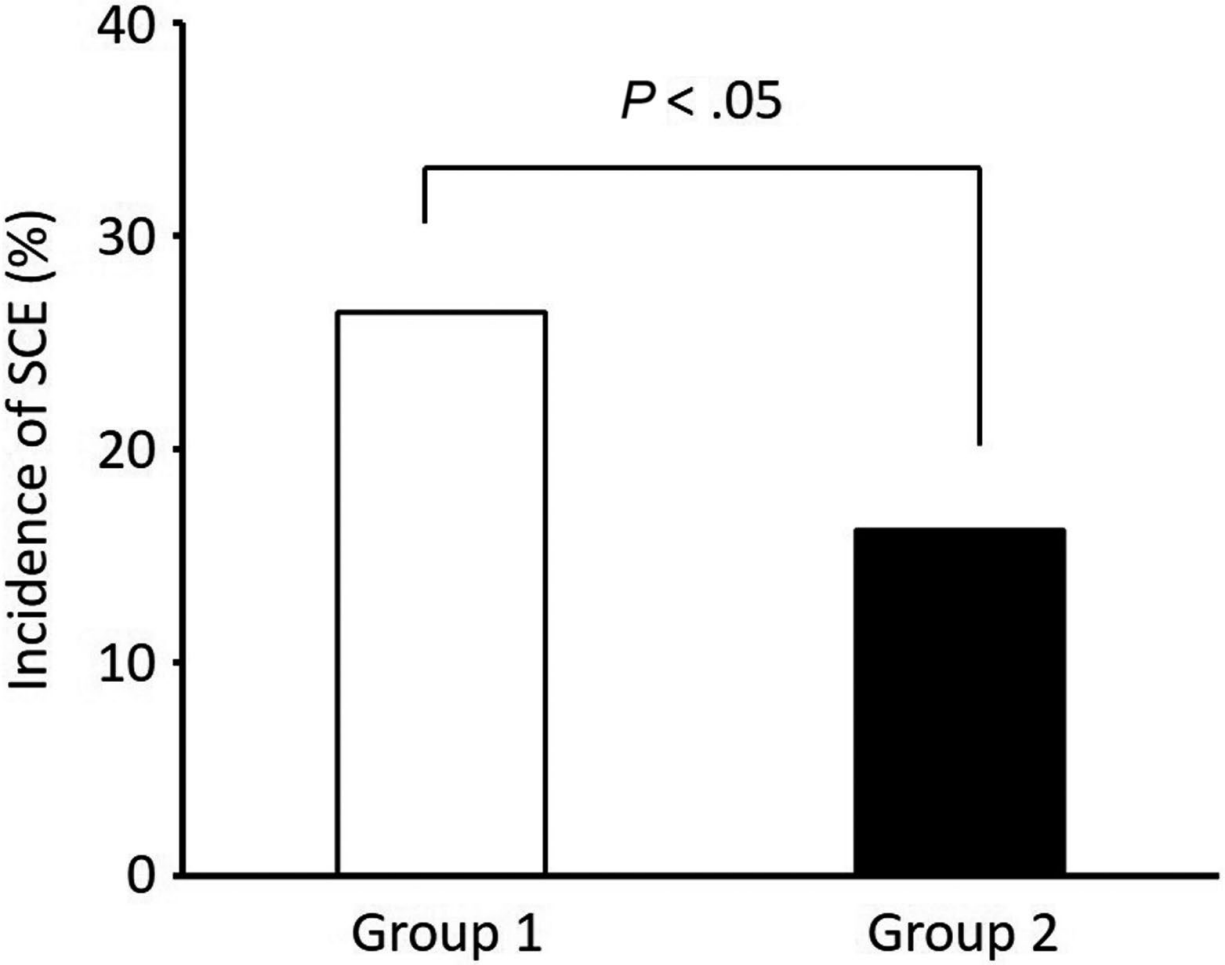
Incidence of silent cerebral events (SCEs)

The incidence of SCE significantly decreased in Group 2 (16.2%), compared to Group 1 (26.4%, Figure 3). Among Group 1, the incidence of SCE was 23% in rivaroxaban, 39% in apixaban, and 19% in edoxaban. Apixaban seems to have higher incidence of SCE than rivaroxaban and edoxaban, but the difference did not reach statistical significance (*P* = .09).

Regarding the number of SCE lesions, 22 patients (61%) had a single lesion whereas 14 (39%) had multiple lesions in Group 1. In Group 2, 15 patients (68%) had single lesion whereas 7 (32%) had multiple lesions. The average number of SCE lesions was comparable between the two groups (1.6 ± 1.0 for Group 1 vs 1.2 ± 0.5 for Group 2, *P* = .08).

We also examined the incidence of SCE in patients taking dabigatran for periprocedural period; dabigatran was uninterruptedly used during the procedure (uninterrupted dabigatran group). The incidence of SCE was compared between Group 2 and the uninterrupted dabigatran group. After propensity score matching, 61 patients in each group were selected for analysis. Patient characteristics after propensity score matching were shown in Table S1. Uninterrupted dabigatran group had lower incidence of SCE than Group 2 (18% vs 23%) but no statistical difference was observed (*P* = .50); “dabigatran bridge” and uninterrupted dabigatran therefore have a similar impact on SCE.

### 3.4 Follow‐up complications

All patients were followed up at least for 30 days and none of them dropped out. Two major bleeding events were observed in Group 1 but none in Group 2. One major event in Group 1 was intracranial hemorrhage (epidural hematoma) in a patient with uninterrupted rivaroxaban (15 mg), causing death. This happened 1 week after discharge although brain MRI (within 2 days after the procedure) showed no sign of intracranial bleeding. Another major bleeding in Group 1 was pseudoaneurysm at the puncture site in a patient with uninterrupted apixaban (10 mg), requiring surgical treatment and blood transfusion. No cardiac tamponade was observed in either group. Six patients in Group 1 (4%) had minor bleeding events: four groin hematomas and two nasal bleeds. Five patients in Group 2 (4%) had minor bleeding: two groin hematomas, two nasal bleeds, and one urinary tract bleed. No thromboembolic events were observed in either group. There was no significant difference between the two groups in the prevalence of major and minor bleeding events.

## 4 DISCUSSION

This study compared uninterrupted DOACs with and without switching to dabigatran (“dabigatran bridge”) in patients taking factor Xa inhibitors prior to AF ablation. The results demonstrated that uninterrupted DOACs with “dabigatran bridge” showed higher initial ACT value, shorter time to achieve ACT > 300 s, and lower incidence of MRI‐detected SCE than uninterrupted DOACs without “dabigatran bridge” (uninterrupted factor Xa inhibitors). However, follow‐up complications between the two groups were comparable.

### 4.1 Comparison with previous studies

Recent studies have evaluated the effectiveness and safety of “dabigatran bridge” for AF ablation by comparing with minimally‐interrupted DOACs strategy.^10^, ^11^ On the other hand, DOACs were uninterruptedly used during the procedure in both groups of our study. Because a recent guideline/expert consensus statement recommends uninterrupted use of warfarin and DOACs for the periprocedural period,^7^ the comparison between different uninterrupted DOACs strategies might be an advantage of this study. After hospital discharge, dabigatran was switched back to the original DOAC prescribed before the procedure in previous studies. Contrarily, the switched dabigatran was continued at least for the follow‐up period in this study. A previous study assessed adverse events for 8 weeks^10^ and the 30‐day follow‐up in this study might not be sufficient for evaluation.

Intraprocedural use of UFH and its monitoring with ACT are important to avoid serious complications. These are affected by the periprocedural OAC strategy.^15^ Microthrombus can form immediately after insertion of a sheath/catheter. Therefore, it is better for ACT to be increased up to the optimal therapeutic range as quickly as possible. Pharmacological impact of direct thrombin inhibitor (dabigatran) on the coagulation cascade is different from factor Xa inhibitors (rivaroxaban/apixaban/edoxaban); dabigatran has more impact on activated partial thromboplastin time (aPTT), mainly related to the activity of intrinsic pathway, than factor Xa inhibitors.^18^ ACT is also related to the intrinsic pathway activity and the use of dabigatran on the day of the procedure would increase initial ACT value, shortening the time to optimal ACT value, which may prevent microthrombus formation during the procedure. In this study, different intraprocedure ACT kinetics between the groups may, at least partly, contribute to the difference in the incidence of SCE, however the mechanisms why “dabigatran bridge” just on the day of the procedure decreased SCE still remains unknown.

SCE is potentially associated with unfavorable neuropsychological outcomes. Strategies to reduce SCE might be advantageous. SCE has been observed with a range from 4.3% to 37.5% in patients undergoing AF ablation.^19^, ^20^, ^21^, ^22^ Several mechanisms of SCE after AF ablation are proposed, such as gaseous emboli and microthrombus. The incidence varies among studies and is affected by multiple factors, therefore we performed propensity score matching and adjusted confounders potentially related to thromboembolism underlying SCE. No previous studies on SCE have done the statistical adjustments. However, unmeasured procedure‐related factors, such as ablation settings (contact force, the number of ablation point, power, irrigation flow rate, etc), type of ablation catheter, procedure time, and operators, might still affect SCEs, and therefore care should be taken when interpreting the result.

Among DOACs, regular dose of dabigatran (150 mg b.i.d.), but not the other DOACs, showed superiority to VKA in the prevention of ischemic stroke in AF patients.^23^ We reported that left atrial appendage thrombus detected by TEE disappeared after treatment with dabigatran in AF patients undergoing catheter ablation,^24^ suggesting that dabigatran potentiates the antithrombotic effect that may prevent the formation of microthrombus during AF ablation. Although no thromboembolic events were observed in either group, the lower incidence of SCE in Group 2 suggests the potential benefit to minimize procedure‐related thromboembolic risk.

Recent studies demonstrated that uninterrupted dabigatran, but not factor Xa inhibitors, decreased the incidence of major bleeding during the periprocedural period of AF ablation when compared to uninterrupted VKA. Although there were more bleeding events in Group 1 than in Group 2, there was no significant difference in the prevalence of follow‐up complications in this study; the clinical efficacy and safety were statistically comparable between the two groups. This is likely attributable to the small number of patients. Nevertheless, the use of dabigatran on the day of AF ablation still seems to have an advantage in case of procedure‐related adverse bleeding in the countries where idarucizumab, but not andexanet alfa, is clinically available.

### 4.2 Limitation

This is a nonrandomized single‐center study of a small number of patients. Treatment adherence of DOACs was not carefully evaluated, and poor adherence in some patients could have caused inadequate anticoagulation. The regular dose of rivaroxaban in Japan is different from that in Europe/North America. However, patient characteristics between the two groups in this study were well‐matched by propensity score, and represent average age, BMI, and CHADS2 score typical of Japanese AF patients undergoing catheter ablation; the results may be more applicable to Japanese patients. Acute SCE lesions reportedly regress during the follow‐up period, but we did not perform brain MRIs during the chronic phase. We performed diffusion‐weighted MRI (DWI) imaging with ADC map but did not perform T2‐weighted axial fluid‐attenuated inversion recovery (FLAIR) imaging, which is a potential drawback of this study. A lack of MRI imaging prior to the procedure is also one of the limitations.

## 5 CONCLUSIONS

Uninterrupted DOACs with “dabigatran bridge” minimizes the preclinical thromboembolic events with similar bleeding risk to uninterrupted factor Xa inhibitors. However, it still has an advantage in case of procedure‐related adverse bleeding in the countries without Andexanet alfa.

## CONFLICT OF INTEREST

MH received speaker fees from Nippon Boehringer Ingelheim and Bristol‐Myers Squibb. EW received research funds from Daiichi‐Sankyo and received speaker fee from Bristol‐Myers Squibb and Biotronik Japan. YM, YN, AN, MK, KM, YO, and YO have nothing to disclose.

## Supporting information

Table S1
