# Supplementary material for: Comparison of effectiveness and safety between uninterrupted direct oral anticoagulants with and without switching to dabigatran in atrial fibrillation ablation
Source: J Arrhythm. 2020 Mar 18;36(3):417–24. doi: 10.1002/joa3.12333 (PMC7280006; doi:10.1002/joa3.12333)
Supplement: Supplementary file 1 — Table S1 [file JOA3-36-417-s001.docx]

**Supplemental Table 1. Patient characteristics in uninterrupted dabigatran group and Group 2 after propensity score matching**

|  | After Propensity Score Matching | | |
| --- | --- | --- | --- |
|  | Uninterrupted Dabigatran Group  (n=61) | Group 2  (n=61) | P value |
| Age, y | 62.0±11.2 | 61.9±12.8 | 0.970 |
| Male, n (%) | 46 (75) | 47 (77) | 0.832 |
| BMI, kg/m^2^ | 24.9±4.5 | 23.9±3.3 | 0.154 |
| Persistent AF, n (%) | 41 (67) | 38 (62) | 0.570 |
| CHADS_2_ score (pts) | 1.18±1.23 | 1.10±1.23 | 0.714 |
| CHA_2_DS_2_-VASc score (pts) | 1.98±1.57 | 1.82±1.57 | 0.714 |
| CHF, n (%) | 19 (31) | 15 (25) | 0.419 |
| HT, n (%) | 26 (43) | 25 (41) | 0.854 |
| Age ≥75, n (%) | 5 (8) | 10 (16) | 0.165 |
| DM, n (%) | 10 (16) | 9 (15) | 0.803 |
| Stroke/TIA, n (%) | 6 (10) | 4 (7) | 0.508 |
| Vascular disease, n (%) | 2 (3) | 4 (7) | 0.410 |
| Blood test and UCG |  |  |  |
| Cr, mg/dl | 0.85±0.16 | 0.82±0.18 | 0.363 |
| CrCl, ml/min | 90.6±36.2 | 90.6±29.6 | 0.993 |
| BNP, pg/ml | 118±117 | 156±210 | 0.239 |
| EF, % | 56.0±7.4 | 54.3±11.8 | 0.340 |
| LAD, mm | 40.4±6.2 | 39.2±6.7 | 0.308 |
| Medication |  |  |  |
| β-blocker, n (%) | 36 (59) | 35 (57) | 0.854 |
| Antiplatelets, n (%) | 1 (2) | 4 (7) | 0.157 |
| AAD, n (%) | 7 (11) | 8 (13) | 0.783 |
| Low dose NOAC, n (%) | 7 (11) | 9 (15) | 0.591 |
| Ablation Procedure |  |  |  |
| Cryoballoon, n (%) | 9 (15) | 9 (15) | 1.000 |
| Additional linear ablation, n (%) | 17 (28) | 11 (18) | 0.200 |

AAD: anti-arrhythmic drug, ACT: activated clotting time, AF: atrial fibrillation, BMI: body mass index, CHF: congestive heart failure, Cr: creatinine, CrCl: creatinine clearance, DM: diabetes mellitus, EF: ejection fraction, HT: hypertension, LAD: left atrial diameter, SCE: silent cerebral event, TE: thromboembolism, TIA: transient ischemic attack, UCG: ultrasonic echocardiography UFH: unfractionated heparin.
